# Vascular relaxation of canine visceral arteries after ischemia by means of supraceliac aortic cross-clamping followed by reperfusion

**DOI:** 10.1186/1757-7241-18-41

**Published:** 2010-07-19

**Authors:** José G Ciscato, Verena K Capellini, Andrea C Celotto, Caroline F Baldo, Edwaldo E Joviliano, Paulo RB Evora, Marcelo B Dalio, Carlos E Piccinato

**Affiliations:** 1 Department of Surgery and Anatomy, School of Medicine of Ribeirão Preto, University of São Paulo, Ribeirão Preto, Brazil

## Abstract

**Background:**

The supraceliac aortic cross-clamping can be an option to save patients with hipovolemic shock due to abdominal trauma. However, this maneuver is associated with ischemia/reperfusion (I/R) injury strongly related to oxidative stress and reduction of nitric oxide bioavailability. Moreover, several studies demonstrated impairment in relaxation after I/R, but the time course of I/R necessary to induce vascular dysfunction is still controversial. We investigated whether 60 minutes of ischemia followed by 30 minutes of reperfusion do not change the relaxation of visceral arteries nor the plasma and renal levels of malondialdehyde (MDA) and nitrite plus nitrate (NOx).

**Methods:**

Male mongrel dogs (n = 27) were randomly allocated in one of the three groups: sham (no clamping, n = 9), ischemia (supraceliac aortic cross-clamping for 60 minutes, n = 9), and I/R (60 minutes of ischemia followed by reperfusion for 30 minutes, n = 9). Relaxation of visceral arteries (celiac trunk, renal and superior mesenteric arteries) was studied in organ chambers. MDA and NOx concentrations were determined using a commercially available kit and an ozone-based chemiluminescence assay, respectively.

**Results:**

Both acetylcholine and calcium ionophore caused relaxation in endothelium-intact rings and no statistical differences were observed among the three groups. Sodium nitroprusside promoted relaxation in endothelium-denuded rings, and there were no inter-group statistical differences. Both plasma and renal concentrations of MDA and NOx showed no significant difference among the groups.

**Conclusion:**

Supraceliac aortic cross-clamping for 60 minutes alone and followed by 30 minutes of reperfusion did not impair relaxation of canine visceral arteries nor evoke biochemical alterations in plasma or renal tissue.

## Background

Traumatic injuries still constitute one of the leading causes of death in all age groups [1]. Intrathoracic or subdiaphragmatic haemorrhage due to trauma is a life-threatening injury and the management of the massive haemorrhage is a great challenge in acute trauma care that often requires emergency surgical repair [1-3]. The supraceliac aortic cross-clamping can be an option to save critical patients with hipovolemic shock due to abdominal trauma [4,5]. However, this maneuver is associated with various complications including myocardial dysfunction, pulmonary disease, renal insufficiency/failure, liver failure, ischemic enterocolitis, coagulopathy and paraparesis/paraplegia [4,6]. Multiple organ failure results from the ischemia and reperfusion (I/R) injury [6], an universal phenomenon that has been extensively studied.

The I/R injury is characterized by an increase in circulating mediators, such as free reactive oxygen metabolites, and cytokines, which reduce the nitric oxide (NO) bioavailability, activate adhesion molecules and neutrophils, and promote lipid peroxidation, impairing the endothelial function [7]. Several investigations have demonstrated reduction in endothelium-dependent relaxation in coronary [8], pulmonary [9], mesenteric [10], renal [11] and femoral [12] arteries submitted to I/R.

Despite a good understanding about the pathogenesis of I/R injury, the time course of I/R necessary to induce vascular dysfunction is still controversial [12-15]. This fact motivated us to investigate whether 60 minutes of ischemia (simulating the clinical time of supraceliac aortic cross-clamping for surgical control of bleeding) followed by 30 minutes of reperfusion do not change the endothelium-dependent and -independent relaxation of visceral arteries nor the plasma and renal levels of malondialdehyde (MDA, an index of lipid peroxidation) and nitrite plus nitrate (NOx). If our hypothesis is true, efforts should be made to establish an effective treatment protocol to prevent the organ failure in this period.

## Methods

### Animal preparation and experimental design

All experimental procedures and animals handling were reviewed and approved by the Institutional Committee for Animal Care and Use of the School of Medicine of Ribeirão Preto, University of São Paulo.

Twenty-seven male mongrel dogs from kennel of School of Medicine of Ribeirão Preto (18-25 kg, young adult) were studied. After an overnight fast except for *ad libitum *water, the animals were premedicated with intramuscular injection of ketamine (15 mg/Kg, Ketamin - S(+), Cristália Produtos Químicos Ltda, Itapira, SP, Brazil) associated with xylazine (2 mg/Kg, Dopaser^®^, Hertape Calier Saúde Animal S/A, Juatuba, MG, Brazil). The anesthesia was maintained with intravenous bolus administration of sodium thiopental (30 mg/kg, Thiopentax, Cristália Produtos Químicos Ltda, Itapira, SP, Brazil). The animals were intubated with an endotracheal tube (8.0 mm, Rüsch, Teleflex Medical, Durham, NC, USA) and ventilated with 100% O_2 _in a pressure controlled mode (Takaoka 600, K. Takaoka Indústria e Comércio Ltda, São Bernardo do Campo, SP, Brazil). An intravenous catheter was placed in the jugular vein for fluid administration and blood drawn. Maintenance fluid consisted of physiologic solution (NaCl 0.9%) at 2 ml/kg/hr and the blood was drawn at end of the experiment (immediately before the euthanasia) for biochemical assessment. The right carotid artery was cannulated for continuous intra-arterial blood pressure and an electrocardiogram monitor showed the heart rate. A median transperitoneal laparotomy was performed and the abdominal supraceliac aorta was exposed. Then, the animals were randomly allocated in one of the three groups: sham (no clamping, n = 9), ischemia (clamping for 60 minutes, n = 9), and ischemia/reperfusion (clamping for 60 minutes followed by reperfusion for 30 minutes, n = 9). The vascular clamp was applied to the abdominal supraceliac aorta except for sham group. The sham animals were submitted to the same surgical procedures with the omission of vascular occlusion and monitored for 90 minutes. After the desirable protocol for each group, the animals were sacrificed with an overdose of sodium thiopental followed by exsanguinations via carotid. Then, the celiac trunk, renal and superior mesenteric arteries were quickly harvested for vascular reactivity studies. Renal tissue samples were also collected. Plasma and renal samples were stored at -70°C until determination of malondialdehyde (MDA) and nitrite and nitrate (NOx) levels.

### Vessel preparation and isometric tension recording

The arterial segments (celiac trunk, renal and superior mesenteric) were carefully dissected free of connective tissue and immersed in a cooled and oxygenated Krebs solution (NaCl: 118.0, KCl: 4.7, CaCl_2_: 2.5, KH_2_PO_4_: 1.2, MgSO_4_: 1.66, glucose: 11.1, NaHCO_3_: 25.0 (mM), pH 7.4). The arterial segments were cut in rings of 4-5 mm in length and prepared with great care to avoid touching the intimal surface. In some rings the endothelium was removed by gently rubbing the intimal surface of the blood vessel with a pair of watchmaker's forceps. This procedure removes endothelium but does not affect the ability of the vascular smooth muscle to contract or relax.

The rings were mounted in organ chambers (10 mL) filled with Krebs solution maintained at 37°C and bubbled with 95% O_2_/5% CO_2 _(pH 7.4). Each arterial ring was suspended by two stainless steel clips placed through the lumen. One clip was anchored to the bottom of the organ chamber, while the other was connected to a strain gauge for measurement of the isometric force using Grass FT03 (Grass Instrument Company, Quincy, MA, USA). The rings were placed at an optimal length-tension of 10 g (determined in a pilot study) and allowed to equilibrate for 60 min with the bath fluid being changed every 15 to 20 min.

Endothelial integrity was assessed qualitatively by the degree of relaxation caused by acetylcholine (Ach, 10^-6 ^M; Sigma, St. Louis, MO, USA) in the presence of contractile tone induced by prostaglandin F_2α _(PGF_2α_, 2.10^-6 ^M; Sigma, St. Louis, MO, USA). For studies of endothelium intact vessels, the ring was discarded if relaxation with Ach was not 80% or greater. For studies of endothelium-denuded vessels, rings were discarded if there was any measurable degree of relaxation. Sequentially, each ring was washed and re-equilibrated for 30 min.

Arterial rings were then precontracted with PGF_2α _(2.10^-6 ^M), and cumulative concentration-response curves were obtained after a stable plateau was reached. The receptor-dependent and -independent relaxations were evoked by Ach (10^-10 ^- 3.10^-5 ^M) and calcium ionophore (A23187, 10^-10 ^- 3.10^-5 ^M, Sigma, St. Louis, MO, USA), respectively, both in endothelium-intact rings. The endothelium-independent relaxation was evoked by sodium nitroprusside (SNP, 10^-10 ^- 3.10^-5 ^M; Sigma, St. Louis, MO, USA) in denuded rings. All concentration-response curves were accomplished by pre-incubating the arterial rings with indomethacin (2.10^-5 ^M, an unspecific cyclooxygenase inhibitor; Sigma, St. Louis, MO, USA) for 50 minutes.

The changes in vascular wall tension are expressed as percent of relaxation in relation to the maximal contraction achieved following exposure to PGF_2α_, a convention that corrects inter-animal variability.

### Malondialdehyde (MDA) measurement

Blood samples were collected in tubes containing EDTA (1:20 v/v). After blood centrifugation (3000×g, 10 min, 4°C), plasma aliquots were stored at -70°C until MDA measurement.

Renal tissue samples were wrapped and promptly stored at -70°C. For analysis, the renal samples were homogenized in Tris-HCl (20 mM, pH 7.4, 10% w/v), the homogenate was centrifuged (3000×g, 10 min, 4°C), and the supernatant was used for the assay.

Plasmatic and renal MDA concentration was measured using a commercially available kit (Lipid Peroxidation Assay kit, Calbiochem, San Diego, CA, USA). The assay is based on the ability of a chromogenic agent to react with MDA, yielding a stable chromophore with maximal absorbance at 586 nm. Results are expressed in μM.

### Nitrite and nitrate (NOx) quantification

Blood samples were collected in tubes containing heparin (1:20 v/v). After blood centrifugation (3000×g, 10 min, 4°C), plasma aliquots were stored at -70°C until NOx measurement.

Renal tissue samples were wrapped and promptly stored at -70°C. For analysis, the renal samples were homogenized in Tris-HCl (20 mM, pH 7.4, 10% w/v), the homogenate was centrifuged (3000×g, 10 min, 4°C), and the supernatant was used for measurement of NOx and total protein by means of the modified biuret reaction [16].

Plasma and renal samples were analyzed using an ozone-based chemiluminescence assay. Briefly, the samples were treated with cold ethanol (1:2 v/v for 30 min at -20°C) and centrifuged (4000×g, 10 min). NOx levels were measured by injecting 25 μL of the supernatant in a glass purge vessel containing 0.8% of vanadium (III) in HCl (1 N) at 90°C, which reduces NOx to NO gas. A nitrogen stream was bubbled through the purge vessel containing vanadium (III), then through NaOH (1 N), and then into an NO analyzer (Sievers^® ^Nitric Oxide Analyzer 280, GE Analytical Instruments, Boulder, CO, USA). NOx concentration was calculated from a standard curve (sodium nitrate 0.5, 1.5, 10, and 50 mM). NOx concentration is expressed in μM for plasma and in μM/mg protein for renal samples.

### Statistical analysis

The results are expressed as mean ± standard error of the mean (SEM). The dose-response curves to Ach, A23187 and SNP were performed using molar concentrations of these drugs and the figures show logarithm of molar concentration (log [M]). The concentration-response curves were analyzed using two-way repeated-measures analysis of variance (ANOVA) and Bonferroni post-test, and the concentrations of MDA and NOx were analyzed using one-way ANOVA (Prism 4.0, GraphPad Software Inc., San Diego, CA, USA). Values were considered to be statistically significant at p values less than 0.05.

## Results

### Vascular function

**Figure 1:**
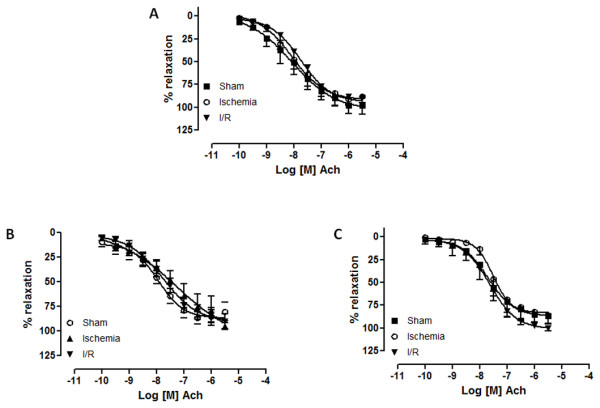
**Concentration-response curves for acetylcholine (10^-10 ^M to 3.10^-5 ^M) in canine celiac trunk (a), superior mesenteric (b) and renal arteries (c) from sham, ischemia and ischemia/reperfusion (I/R) groups**. (n = 9). Log [M] = logarithm of molar concentration.

**Figure 2:**
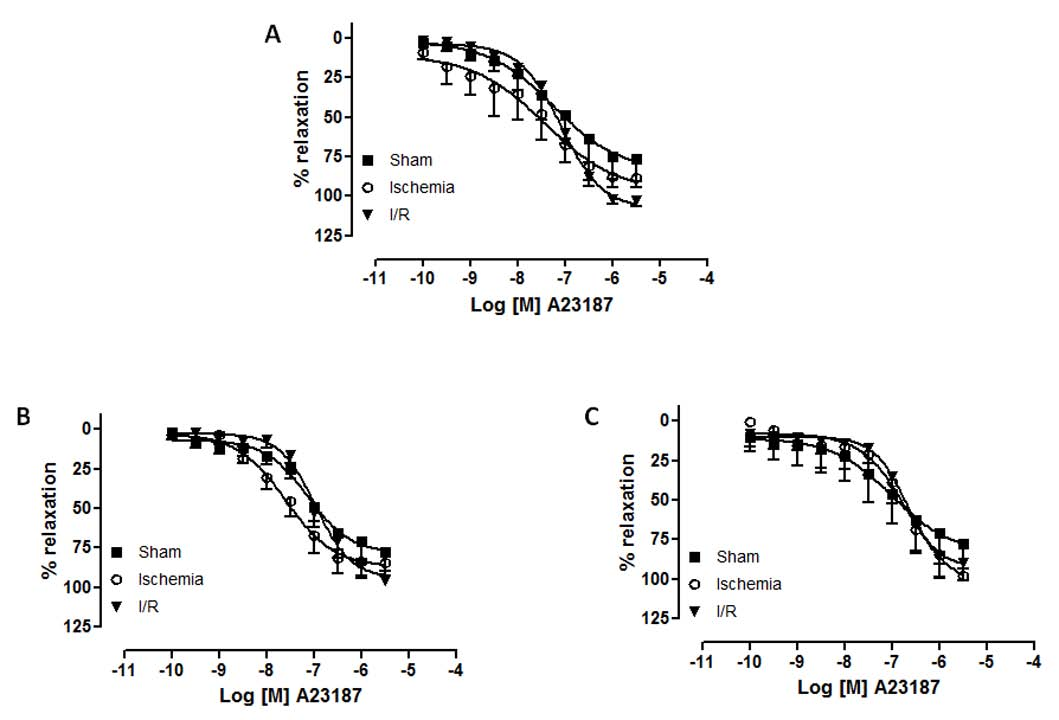
**Concentration-response curves for calcium ionophore (A23187 - 10^-10 ^M to 3.10^-5 ^M) in canine celiac trunk (a), superior mesenteric (b) and renal arteries (c) from sham, ischemia and ischemia/reperfusion (I/R) groups**. (n = 9). Log [M] = logarithm of molar concentration.

Both Ach and A23187 caused concentration-dependent relaxation in endothelium-intact rings of celiac trunk, renal and superior mesenteric arteries and no statistical differences were observed among the three groups (Figures 1 and 2).

**Figure 3:**
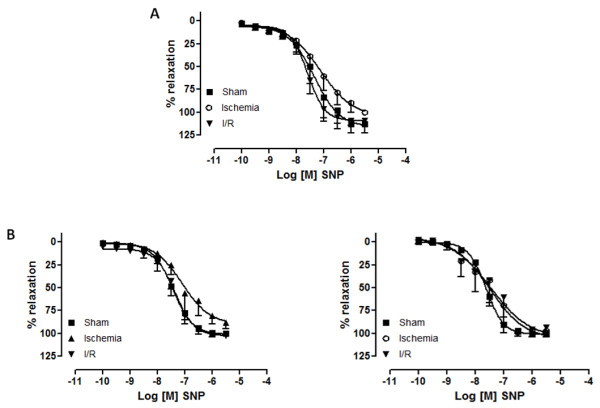
**Concentration-response curves for sodium nitroprusside (10^-10 ^M to 3.10^-5 ^M) in canine celiac trunk (a), superior mesenteric (b) and renal arteries (c) from sham, ischemia and ischemia/reperfusion (I/R) groups**. (n = 9). Log [M] = logarithm of molar concentration.

SNP caused concentration-dependent relaxation in endothelium-denuded rings of the three studied arteries, and there were no inter-group statistical differences (Figure 3).

### Malondialdehyde (MDA) and nitrite/nitrate (NOx) concentrations

Both plasma and renal concentrations of MDA and NOx showed no significant difference among the three groups (Tables 1 and 2).

**Table 1:** Plasmatic concentrations of malondialdehyde (MDA) and nitrite/nitrate (NOx) in sham, ischemia, and ischemia/reperfusion groups

	Sham	Ischemia	Ischemia/reperfusion
MDA (μM)	3.47 ± 0.48	4.05 ± 0.93	5.65 ± 1.85
NOx (μM/)	26.96 ± 3.70	30.17 ± 4.27	37.87 ± 5.44

All values are means ± SEM (n = 9).

**Table 2:** Renal concentrations of malondialdehyde (MDA) and nitrite/nitrate (NOx) in sham, ischemia, and ischemia/reperfusion groups

	Sham	Ischemia	Ischemia/reperfusion
MDA (μM)	5.59 ± 0.90	7.44 ± 0.39	5.38 ± 0.34
NOx (μM/mg protein)	1.09 ± 0.09	1.03 ± 0.14	1.00 ± 0.14

All values are means ± SEM (n = 9).

## Discussion

The results of the present study indicate that 60 minutes of ischemia by means of supraceliac aortic cross-clamping alone or followed by 30 minutes of reperfusion do not affect the endothelium-dependent and -independent relaxation of canine celiac trunk, renal and superior mesenteric arteries. Previous investigations showed controversy results. Koksoy et al. (2000) demonstrated that rabbit abdominal aorta, superior mesenteric, renal, pulmonary, and carotid arteries present unchanged endothelial and smooth muscle function after one-hour intestinal ischemia with two- or four-hour reperfusion [14]. The former group reported that the same model of I/R in rats led to a significant reduction in the ability of the pulmonary vasculature to respond to Ach, and calcium ionophore, but not to nitroglycerin [13]. Sobey et al. (1990) showed that coronary artery occlusion for 60 minutes and reperfusion for 30 minutes attenuate endothelium-dependent and -independent relaxation of canine coronary arteries in vivo, whereas only endothelium-dependent relaxation is inhibited in vitro [15]. Martinez-Revelles et al. (2008) observed impaired Ach vasodilation without modifying the vasodilation to SNP in mesenteric resistance artery obtained from rats submitted to 90 minutes of cerebral ischemia with 24 hours of reperfusion [17]. Joviliano et al. (2005) investigated different times of I/R by means of infrarenal aortic cross-clamping in dogs and concluded that 120 minutes of ischemia alone and 90 minutes of ischemia followed by 60 minutes of reperfusion did not impair the endothelium-dependent relaxation of the femoral artery, whereas 120 minutes of ischemia followed by 90 minutes of reperfusion led to reduced relaxation [12]. Comparing these findings [12] with those of the present study, it can be observed that canine visceral and femoral arteries have similar responses, since shorter times of I/R did not cause significant alterations on endothelium-dependent and -independent relaxation, differently of that observed for coronary arteries [15]. The duration of I/R, the vessel size, and the species and organ-specific differences may be possible explanations for these discrepancies.

It is well known that during ischemia, the cell structures are progressively damaged, but restoration of the blood flow, paradoxically, intensifies the lesions caused by the ischemia. In other words, depending on the time and intensity of ischemia, tissue injury can be further exacerbated in the reperfusion [18]. A clinical study revealed that the visceral ischemic time during supraceliac, above the superior mesenteric artery or suprarenal clamping, and not clamp location, is the only independent predictor of operative mortality and that visceral ischemia time longer than 32 minutes is the strongest predictor of early death [19]. On the other hand, an experimental study with dogs showed that liver ischemic time equal or less than 60 minutes does not induce vascular dysfunction in hepatic artery [20]. Not only the ischemic time, but also the reperfusion time is an important feature in the development of vascular dysfunction. Davenpeck et al. (1993) analyzed the rabbit pulmonary artery following in vivo I/R of the lung, and showed that endothelium-dependent relaxation remained essentially normal after 90 min of ischemia and 30 min of reperfusion, while 90 min of ischemia followed by 60 min of reperfusion resulted in a significant decrease in endothelium-dependent relaxation to A23187, and 90 min of ischemia followed by 90 min of reperfusion resulted in significant attenuation of endothelium-dependent relaxation to both ACh and A23187 [9].

Concerning the vessel size, studies postulated that I/R impairs endothelium-dependent relaxation of microvessels, but does not affect large arteries [14,21]. Quillen et al. found that dogs after 1 hour of coronary artery occlusion with 1 hour of reperfusion had impairment of endothelium-dependent responses in the coronary microcirculation, but not in large epicardial coronary arteries [21].

In the present investigation, ischemia and I/R did not change the plasma and renal levels of MDA and NOx, and according to the pathophysiology of I/R, these results can be due to reperfusion insufficient time. It has been suggested that NO, produced from endothelial nitric oxide synthase (eNOS), may be an important protective molecule at the onset of I/R. However, the I/R induced-cytokines activate the transcription of the inducible nitric oxide synthase (iNOS), which produces large amounts of NO presenting harmful effects. The NO excess reacts with superoxide, originated when oxygen is reintroduced to the ischemic tissue during reperfusion, to produce peroxynitrite. The peroxynitrite promotes lipid peroxidation with consequent cellular damage, and leads to a phenomenon known as "NOS uncoupling", which reduces the NO synthesis and increases the oxidative stress [18,22]. Corroborating our findings, previous studies showed that the increase in oxidative stress and in the concentration of NO metabolities occurs in I/R time longer than the one performed in our protocol [23-25]. Grisotto et al. (2000) observed membrane phospholipid damage after 3 hours of skeletal muscle ischemia with significant oxidative alterations after more 45 minutes of reperfusion [24]. Ozkan et al. (2009) detected severe increases in the tissue levels of MDA and NO in rats subjected to intestinal ischemia (60 min) and subsequent reperfusion (60 min) [25]. Chen et al. (2008) observed enhancement in renal content of MDA and in serum concentration of NO in rats exposed to 45 min of renal ischemia followed by 24 hours of reperfusion [23].

In summary, the supraceliac aortic cross-clamping for 60 minutes alone or followed by 30 minutes of reperfusion in dogs did not impair the relaxation of visceral arteries, neither change the concentration of MDA and NOx in plasma and renal tissue, indicating that during this period, there is still no vascular dysfunction nor evidence of oxidative stress. These results make this period an important opportunity to treat those trauma casualties needing this kind of surgery in order to prevent the I/R injury, and point the need of more studies. We cannot end the discussion without mentioning that although the I/R injury is an important mechanism associated with multiple organ failure in trauma patients or other complex aortic reconstructions, several mechanisms may lead to the development of ischemic complications (mainly renal, hepatic and mesenteric dysfunctions). Therefore, it is important to point that previous subclinical organ failures can interfere individually adding a kind of bias over the main idea of this investigation that considered the supraceliac cross-clamping as a lifesaving trauma maneuver.

## Competing interests

The authors declare that they have no competing interests.

## Authors' contributions

JGCJ has been involved in collecting data, analysis and interpretation of data and drafting the manuscript. VKC has been involved in collecting data, analysis and interpretation of data and drafting the manuscript. ACC has been involved in design the study, collecting data, analysis and interpretation of data and drafting the manuscript. CFB has been involved in collecting data. EEJ helped to drafting the manuscript. MBD helped drafting the manuscript. PRBE participated in the design of the study and analysis and interpretation of data and all experiments were performed in his laboratory. CEP participated in the design of the study, acquisition of funding to develop the study and given final approval of the version to be published. All authors have read and approved the final manuscript.
